# 6-Hy­droxy­imino-5α-cholestane

**DOI:** 10.1107/S1600536812040093

**Published:** 2012-09-29

**Authors:** Shams Uzzaman, Hena Khanam, Ashraf Mashrai, Yahia Nasser Mabkhot, Ahmad Husain

**Affiliations:** a Department of Chemistry, Aligarh Muslim University, Aligarh 202 002, India; b Department of Chemistry, Faculty of Science, King Saud University, Riyadh 11451, Saudi Arabia; c Department of Chemistry, University of Cape Town, Cape Town, South Africa

## Abstract

The title compound, C_27_H_47_NO, is a steroid derivative composed of a saturated carbon fused-ring framework with an alkyl side chain. Ring bond lengths have normal values with an average of 1.533 (2) Å, while the cholestane side chain shows an average bond length of 1.533 (2) Å. The three cyclohexane rings adopt chair conformations or close to chair conformations while the cyclopentane ring is twisted. The cholesterol side-chain is fully extended with a *gauche*–*trans* conformation of the terminal methyl groups. There are eight chiral centres in the molecule; the absolute configuration of these sites was determined from the structure presented. There are two molecules in the asymmetric unit; in one, the alkyl chain is disordered over two sets of sites [occupancy ratios of 0.50:0.50 and 0.67:0.33].

## Related literature

For background on steroidal hormone applications, see: Grover *et al.* (2007 ▶). For background to this study and previous syntheses, see: Shoppee *et al.* (1955 ▶). For related structures, see: Ketuly *et al.* (2011 ▶); Park (2004 ▶). For reference bond-length data, see: Allen *et al.* (1987 ▶). For the stability of the temperature controller used for the data collection, see: Cosier & Glazer (1986 ▶).
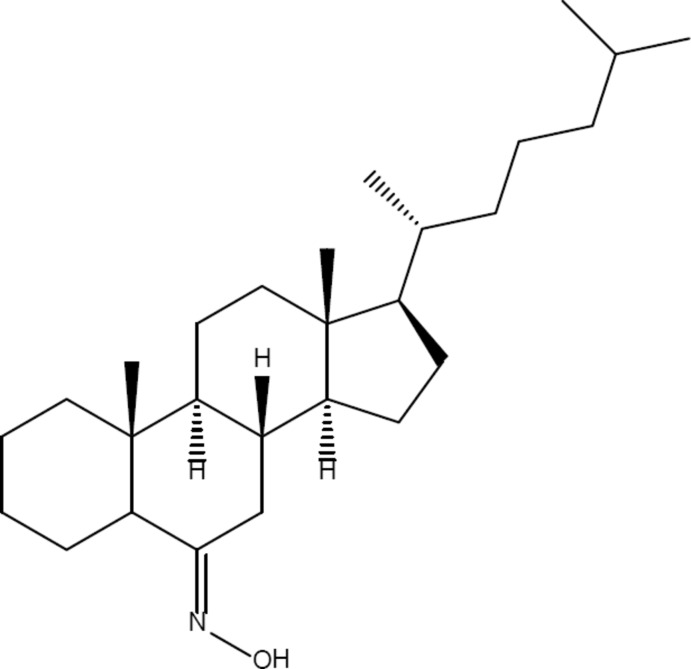


## Experimental

### 

#### Crystal data

C_27_H_47_NO
*M* *_r_* = 401.65
Monoclinic, 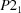

*a* = 13.7535 (7) Å
*b* = 9.5266 (4) Å
*c* = 18.681 (1) Å
β = 102.829 (3)°
*V* = 2386.6 (2) Å^3^
*Z* = 4
Mo *K*α radiation
μ = 0.07 mm^−1^
*T* = 100 K
0.54 × 0.31 × 0.17 mm

#### Data collection

Bruker Kappa APEXII Duo diffractometer
Absorption correction: multi-scan (Blessing, 1995 ▶) *T*_min_ = 0.965, *T*_max_ = 0.989
51587 measured reflections
7706 independent reflections
6021 reflections with *I* > 2σ(*I*)
*R*_int_ = 0.052

#### Refinement

*R*[*F* ^2^ > 2σ(*F* ^2^)] = 0.049
*wR*(*F* ^2^) = 0.129
*S* = 1.12
7706 reflections
564 parameters
59 restraints
H atoms treated by a mixture of independent and constrained refinement
Δρ_max_ = 0.48 e Å^−3^
Δρ_min_ = −0.32 e Å^−3^

### 

Data collection: *APEX2* (Bruker, 2008 ▶); cell refinement: *APEX2*; data reduction: *APEX2*; program(s) used to solve structure: *SHELXS97* (Sheldrick, 2008 ▶); program(s) used to refine structure: *SHELXL97* (Sheldrick, 2008 ▶); molecular graphics: *ORTEP-3* (Farrugia, 1997 ▶); software used to prepare material for publication: *publCIF* (Westrip, 2010 ▶).

## Supplementary Material

Crystal structure: contains datablock(s) I, global. DOI: 10.1107/S1600536812040093/hg5240sup1.cif

Structure factors: contains datablock(s) I. DOI: 10.1107/S1600536812040093/hg5240Isup2.hkl

Additional supplementary materials:  crystallographic information; 3D view; checkCIF report

## Figures and Tables

**Fig. 1.**
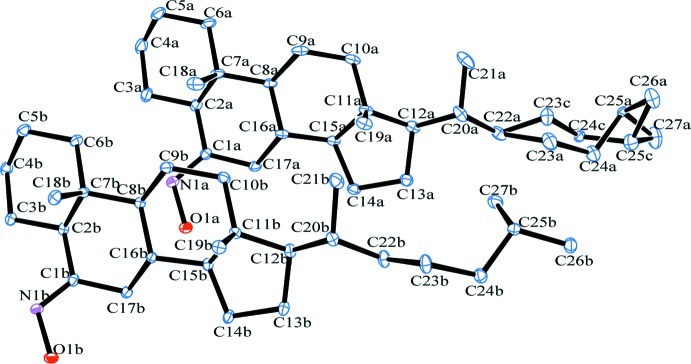
The molecular structure of (I) showing the atomic numbering and 30% probability displacement ellipsoids.

**Fig. 2.**
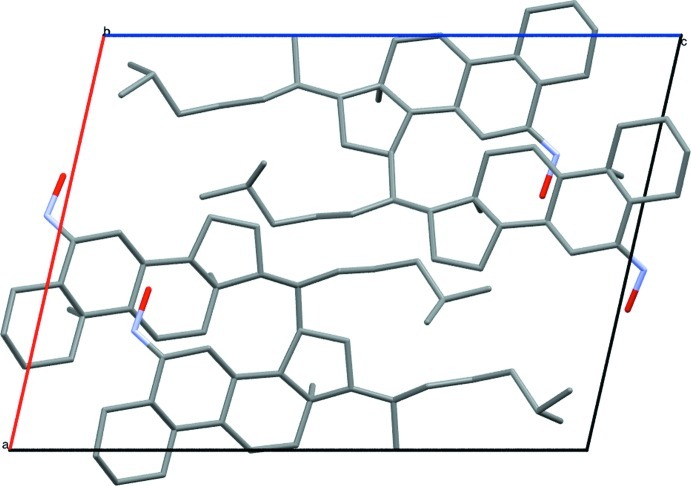
The unit cell diagram projected down the b axis.

## supplementary crystallographic information

### Comment

Steroids are compounds of biological origin and play an important role in biological systems. The dramatic expansion of steroidal chemistry came with the discovery of steroidal hormones. The discovery of several steroids with their wide application in therapy has brought about an increasing interest (Grover *et al.*, 2007). A series of crystal structures of hydroxyiminocholestane (Ketuly *et al.*, 2011) and derivatives of cholesterol (Park, 2004) have been examined in order to obtain structural information relevant to the liquid crystalline phases and the possible modes of association of the cholesterol derivatives themselves.

In the view of the biological importance of cholesterol derivatives, we report here the crystal structure of the title compound (I). There are two molecules in the asymmetric unit (Fig. 1), while unit cell contains 4 molecules (Fig. 2). The ring bond lengths have normal values (Allen *et al.*, 1987) with average C(sp3)—C(sp3) of 1.533 (2) Å, excluding the shorter C1—C62 bond of 1.496 (2) Å. The cholestane side-chain shows C(sp3)—C(sp3) bond lengths varying from C25—C26 [1.525 (2) Å] up to C20—C22 [1.539 (2) Å]. Each of the six-member rings adopts a chair conformation or close to a chair conformation, and the five-member ring has a twisted conformation. There are eight chiral centres in the molecule, the absolute configuration of these sites was determined from the structure presented, these sites exhibit the following chiralities: C2= S, C7 = R, C8 = S, C11 = R, C12 = R, C15 = S, C16 = S and C20 = R.

### Experimental

5α-Cholest-6-one (1.0 g) was dissolved in ethanol (60 ml) and to this, hydroxylamine hydrochloride (1.0 g) and sodium acetate trihydrate (2.0 g) were added. The reaction mixture was refluxed for 4 h. Dilution with cold water gave crude oxime, which was filtered, washed with water and air dried. Recrystallization from ethanol gave crystals of product. m.p. 482 K (reported m.p. 477 K; Shoppee *et al.* 1955). The crystal was placed in the cold stream of an Oxford Cyrosystems Cobra open-flow nitrogen cryostat (Cosier & Glazer, 1986) operating at 100.0 (1) K.

### Refinement

All H atoms were positioned geometrically [C—H = 0.98–1.00 Å] and were refined using a riding model, with *U*_iso_(H) = 1.2–1.5*U*_eq_(C). H atoms attached to O atom were positioned with idealized geometry and were refined isotropically with U_eq_(H) set to 1.5 times of U_eq_(O) using a riding model with O—H = 0.87 Å. In the absence of significant anomalous scattering, Friedel pairs were merged and the absolute structure was assigned arbitrarily. Friedel pairs have been merged using MERG 3, which makes the Flack *x* parameter meaningless and therefore has been removed from the final cif file.

There are two molecules in the asymmetric unit and only alkyl chain (C23*a*—C25*a*) of one molecule is disordered. C23*a* and C25*a* are disordered over two positions with site occupancy factors of 50% while C24*a* is disordered over two positions with site occupancy factors of 33% and 67%.

### Crystal data

**Table:**

C_27_H_47_NO	*F*(000) = 896
*M**_r_* = 401.65	*D*_x_ = 1.118 Mg m^−^^3^
Monoclinic, *P*2_1_	Mo *K*α radiation, λ = 0.71073 Å
Hall symbol: P 2yb	Cell parameters from 10665 reflections
*a* = 13.7535 (7) Å	θ = 1.7–30.6°
*b* = 9.5266 (4) Å	µ = 0.07 mm^−^^1^
*c* = 18.681 (1) Å	*T* = 100 K
β = 102.829 (3)°	Plate, colourless
*V* = 2386.6 (2) Å^3^	0.54 × 0.31 × 0.17 mm
*Z* = 4	

### Data collection

**Table:**

Bruker Kappa APEXII Duo diffractometer	7706 independent reflections
Radiation source: fine-focus sealed tube	6021 reflections with *I* > 2σ(*I*)
Triumph monochromator	*R*_int_ = 0.052
Detector resolution: 0 pixels mm^-1^	θ_max_ = 30.6°, θ_min_ = 1.7°
ω and φ scans	*h* = −18→19
Absorption correction: multi-scan (Blessing, 1995)	*k* = −13→13
*T*_min_ = 0.965, *T*_max_ = 0.989	*l* = −26→26
51587 measured reflections	

### Refinement

**Table:**

Refinement on *F*^2^	Primary atom site location: structure-invariant direct methods
Least-squares matrix: full	Secondary atom site location: difference Fourier map
*R*[*F*^2^ > 2σ(*F*^2^)] = 0.049	Hydrogen site location: inferred from neighbouring sites
*wR*(*F*^2^) = 0.129	H atoms treated by a mixture of independent and constrained refinement
*S* = 1.12	*w* = 1/[σ^2^(*F*_o_^2^) + (0.0568*P*)^2^ + 0.5458*P*] where *P* = (*F*_o_^2^ + 2*F*_c_^2^)/3
7706 reflections	(Δ/σ)_max_ = 0.005
564 parameters	Δρ_max_ = 0.48 e Å^−^^3^
59 restraints	Δρ_min_ = −0.32 e Å^−^^3^

### Special details

**Table:**

Geometry. All e.s.d.'s (except the e.s.d. in the dihedral angle between two l.s. planes) are estimated using the full covariance matrix. The cell e.s.d.'s are taken into account individually in the estimation of e.s.d.'s in distances, angles and torsion angles; correlations between e.s.d.'s in cell parameters are only used when they are defined by crystal symmetry. An approximate (isotropic) treatment of cell e.s.d.'s is used for estimating e.s.d.'s involving l.s. planes.
Refinement. Refinement of *F*^2^ against ALL reflections. The weighted *R*-factor *wR* and goodness of fit *S* are based on *F*^2^, conventional *R*-factors *R* are based on *F*, with *F* set to zero for negative *F*^2^. The threshold expression of *F*^2^ > σ(*F*^2^) is used only for calculating *R*-factors(gt) *etc*. and is not relevant to the choice of reflections for refinement. *R*-factors based on *F*^2^ are statistically about twice as large as those based on *F*, and *R*-factors based on ALL data will be even larger.

### Fractional atomic coordinates and isotropic or equivalent isotropic displacement parameters (Å^2^)

**Table:**

	*x*	*y*	*z*	*U*_iso_*/*U*_eq_	Occ. (<1)
O1A	0.61310 (13)	0.1041 (2)	0.17724 (10)	0.0271 (4)	
H1A	0.585 (2)	0.091 (4)	0.1311 (19)	0.041*	
C1A	0.77678 (17)	0.0812 (2)	0.22893 (13)	0.0190 (4)	
N1A	0.71254 (15)	0.0947 (2)	0.16853 (11)	0.0223 (4)	
C2A	0.88428 (18)	0.0668 (3)	0.22606 (13)	0.0217 (5)	
H2A	0.9058	−0.0269	0.2481	0.026*	
C3A	0.9035 (2)	0.0630 (3)	0.14838 (14)	0.0307 (6)	
H3A1	0.8627	−0.0120	0.1196	0.037*	
H3A2	0.8836	0.1537	0.1236	0.037*	
C4A	1.0132 (2)	0.0357 (4)	0.15124 (16)	0.0403 (8)	
H4A1	1.0317	−0.0584	0.1723	0.048*	
H4A2	1.0250	0.0373	0.1008	0.048*	
C5A	1.0775 (2)	0.1455 (4)	0.19742 (17)	0.0402 (8)	
H5A1	1.0633	0.2384	0.1736	0.048*	
H5A2	1.1486	0.1235	0.2002	0.048*	
C6A	1.05897 (18)	0.1526 (3)	0.27529 (16)	0.0296 (6)	
H6A1	1.0996	0.2296	0.3023	0.036*	
H6A2	1.0818	0.0637	0.3011	0.036*	
C7A	0.94860 (17)	0.1770 (3)	0.27754 (15)	0.0216 (5)	
C8A	0.93347 (16)	0.1497 (2)	0.35607 (13)	0.0183 (4)	
H8A	0.9522	0.0493	0.3672	0.022*	
C9A	1.00016 (18)	0.2365 (3)	0.41720 (15)	0.0260 (5)	
H9A1	1.0707	0.2227	0.4149	0.031*	
H9A2	0.9842	0.3372	0.4084	0.031*	
C10A	0.98758 (18)	0.1972 (3)	0.49393 (15)	0.0251 (5)	
H10A	1.0290	0.2607	0.5303	0.030*	
H10B	1.0121	0.1003	0.5053	0.030*	
C11A	0.87903 (17)	0.2066 (2)	0.50136 (13)	0.0205 (5)	
C12A	0.85108 (17)	0.1293 (3)	0.56717 (13)	0.0218 (5)	
H12A	0.8823	0.0341	0.5697	0.026*	
C13A	0.73665 (18)	0.1076 (3)	0.54132 (13)	0.0255 (5)	
H13A	0.7170	0.0157	0.5584	0.031*	
H13B	0.7003	0.1825	0.5612	0.031*	
C14A	0.71249 (17)	0.1130 (3)	0.45644 (13)	0.0227 (5)	
H14A	0.6756	0.0282	0.4351	0.027*	
H14B	0.6726	0.1973	0.4380	0.027*	
C15A	0.81518 (16)	0.1194 (2)	0.43825 (13)	0.0188 (4)	
H15A	0.8422	0.0217	0.4451	0.023*	
C16A	0.82378 (16)	0.1631 (2)	0.36144 (13)	0.0185 (4)	
H16A	0.8028	0.2634	0.3534	0.022*	
C17A	0.75460 (17)	0.0716 (3)	0.30388 (13)	0.0213 (5)	
H17A	0.7611	−0.0275	0.3203	0.026*	
H17B	0.6847	0.1007	0.3008	0.026*	
C18A	0.9167 (2)	0.3268 (3)	0.25160 (17)	0.0309 (6)	
H18A	0.9526	0.3948	0.2873	0.046*	
H18B	0.8448	0.3374	0.2473	0.046*	
H18C	0.9326	0.3436	0.2037	0.046*	
C19A	0.84505 (19)	0.3604 (3)	0.49939 (15)	0.0243 (5)	
H19A	0.8492	0.4024	0.4523	0.036*	
H19B	0.8883	0.4123	0.5394	0.036*	
H19C	0.7760	0.3646	0.5052	0.036*	
C20A	0.8800 (2)	0.1919 (3)	0.64513 (14)	0.0262 (5)	
H20A	0.8443	0.2833	0.6453	0.031*	
C21A	0.9927 (2)	0.2192 (3)	0.66857 (16)	0.0366 (7)	
H21A	1.0102	0.2433	0.7209	0.055*	
H21B	1.0107	0.2971	0.6398	0.055*	
H21C	1.0290	0.1346	0.6600	0.055*	
C22A	0.8456 (2)	0.0920 (3)	0.69885 (14)	0.0283 (6)	
H22A	0.8951	0.0151	0.7104	0.034*	
H22B	0.7818	0.0493	0.6730	0.034*	
C23A	0.8299 (5)	0.1538 (7)	0.7740 (3)	0.0367 (14)	0.50
H23A	0.8899	0.2111	0.7940	0.044*	0.50
H23B	0.7736	0.2206	0.7609	0.044*	0.50
C24A	0.8103 (6)	0.0677 (10)	0.8393 (4)	0.0321 (18)	0.33
H24A	0.7725	−0.0175	0.8195	0.039*	0.33
H24B	0.7669	0.1240	0.8640	0.039*	0.33
C25A	0.9059 (3)	0.0193 (5)	0.9001 (2)	0.0192 (9)	0.50
H25A	0.9619	−0.0205	0.8803	0.023*	0.50
C23C	0.8831 (5)	0.1306 (6)	0.7778 (3)	0.0309 (12)	0.50
H23C	0.8570	0.2238	0.7875	0.037*	0.50
H23D	0.9569	0.1354	0.7895	0.037*	0.50
C24C	0.8488 (3)	0.0208 (4)	0.82512 (18)	0.0195 (7)	0.67
H24C	0.7828	−0.0124	0.7978	0.023*	0.67
H24D	0.8951	−0.0597	0.8282	0.023*	0.67
C25C	0.8391 (4)	0.0519 (6)	0.9022 (3)	0.0339 (12)	0.50
H25C	0.7727	0.0940	0.9029	0.041*	0.50
C26A	0.9295 (3)	0.1526 (3)	0.94258 (16)	0.0392 (7)	
H26D	0.9898	0.1411	0.9811	0.059*	0.50
H26E	0.9417	0.2269	0.9105	0.059*	0.50
H26F	0.8759	0.1782	0.9645	0.059*	0.50
H26G	0.9264	0.2414	0.9176	0.059*	0.50
H26H	0.9261	0.1672	0.9925	0.059*	0.50
H26I	0.9938	0.1094	0.9417	0.059*	0.50
C27A	0.8563 (3)	−0.0864 (3)	0.94673 (17)	0.0457 (8)	
H27D	0.9057	−0.1284	0.9857	0.069*	0.50
H27E	0.8078	−0.0387	0.9680	0.069*	0.50
H27F	0.8231	−0.1605	0.9151	0.069*	0.50
H27G	0.8061	−0.1545	0.9264	0.069*	0.50
H27H	0.9211	−0.1246	0.9457	0.069*	0.50
H27I	0.8534	−0.0668	0.9964	0.069*	0.50
O1B	0.33906 (12)	0.5733 (2)	−0.01531 (10)	0.0226 (4)	
H1B	0.306 (2)	0.588 (4)	−0.0599 (18)	0.034*	
C1B	0.49582 (17)	0.4940 (2)	0.02841 (12)	0.0170 (4)	
N1B	0.43489 (14)	0.5418 (2)	−0.02822 (10)	0.0178 (4)	
C2B	0.59958 (17)	0.4588 (2)	0.02224 (12)	0.0166 (4)	
H2B	0.6128	0.3617	0.0423	0.020*	
C3B	0.61520 (18)	0.4535 (3)	−0.05631 (12)	0.0203 (5)	
H3B1	0.5658	0.3891	−0.0861	0.024*	
H3B2	0.6048	0.5481	−0.0786	0.024*	
C4B	0.72015 (18)	0.4031 (3)	−0.05655 (13)	0.0222 (5)	
H4B1	0.7304	0.4051	−0.1074	0.027*	
H4B2	0.7283	0.3049	−0.0388	0.027*	
C5B	0.79807 (19)	0.4957 (3)	−0.00758 (14)	0.0233 (5)	
H5B1	0.7953	0.5911	−0.0289	0.028*	
H5B2	0.8653	0.4569	−0.0058	0.028*	
C6B	0.78092 (17)	0.5050 (3)	0.07050 (13)	0.0216 (5)	
H6B1	0.8308	0.5699	0.0995	0.026*	
H6B2	0.7920	0.4112	0.0937	0.026*	
C7B	0.67551 (16)	0.5563 (2)	0.07400 (12)	0.0163 (4)	
C8B	0.65899 (16)	0.5382 (2)	0.15342 (12)	0.0159 (4)	
H8B	0.6737	0.4376	0.1670	0.019*	
C9B	0.73071 (17)	0.6262 (3)	0.21092 (13)	0.0198 (5)	
H9B1	0.7220	0.7265	0.1971	0.024*	
H9B2	0.8001	0.5997	0.2102	0.024*	
C10B	0.71515 (17)	0.6082 (3)	0.28966 (12)	0.0197 (5)	
H10C	0.7340	0.5115	0.3067	0.024*	
H10D	0.7595	0.6740	0.3228	0.024*	
C11B	0.60703 (16)	0.6356 (2)	0.29422 (12)	0.0156 (4)	
C12B	0.57603 (17)	0.5883 (2)	0.36572 (12)	0.0182 (4)	
H12B	0.6061	0.4937	0.3793	0.022*	
C13B	0.46118 (18)	0.5684 (3)	0.34010 (12)	0.0200 (5)	
H13C	0.4393	0.4856	0.3644	0.024*	
H13D	0.4258	0.6522	0.3526	0.024*	
C14B	0.43838 (18)	0.5467 (3)	0.25580 (12)	0.0199 (5)	
H14C	0.4000	0.4593	0.2417	0.024*	
H14D	0.4001	0.6269	0.2300	0.024*	
C15B	0.54085 (16)	0.5372 (2)	0.23794 (11)	0.0156 (4)	
H15B	0.5655	0.4396	0.2504	0.019*	
C16B	0.54998 (16)	0.5646 (2)	0.15896 (11)	0.0149 (4)	
H16B	0.5341	0.6657	0.1475	0.018*	
C17B	0.47601 (17)	0.4750 (3)	0.10383 (12)	0.0188 (5)	
H17C	0.4837	0.3749	0.1182	0.023*	
H17D	0.4069	0.5040	0.1038	0.023*	
C18B	0.66091 (19)	0.7094 (2)	0.04869 (14)	0.0215 (5)	
H18D	0.7133	0.7676	0.0786	0.032*	
H18E	0.5955	0.7425	0.0543	0.032*	
H18F	0.6646	0.7160	−0.0030	0.032*	
C19B	0.57851 (18)	0.7902 (2)	0.27894 (13)	0.0191 (4)	
H19D	0.6164	0.8486	0.3186	0.029*	
H19E	0.5070	0.8021	0.2762	0.029*	
H19F	0.5939	0.8185	0.2322	0.029*	
C20B	0.60358 (18)	0.6827 (2)	0.43447 (12)	0.0198 (5)	
H20B	0.5677	0.7740	0.4226	0.024*	
C21B	0.71555 (19)	0.7145 (3)	0.45636 (14)	0.0264 (5)	
H21D	0.7307	0.7631	0.5038	0.040*	
H21E	0.7344	0.7743	0.4190	0.040*	
H21F	0.7532	0.6264	0.4603	0.040*	
C22B	0.5687 (2)	0.6171 (3)	0.49985 (13)	0.0235 (5)	
H22C	0.5019	0.5749	0.4820	0.028*	
H22D	0.6153	0.5410	0.5211	0.028*	
C23B	0.5635 (2)	0.7249 (3)	0.55994 (13)	0.0257 (5)	
H23E	0.6270	0.7778	0.5710	0.031*	
H23F	0.5097	0.7925	0.5399	0.031*	
C24B	0.54498 (18)	0.6672 (3)	0.63243 (13)	0.0223 (5)	
H24E	0.5053	0.7372	0.6529	0.027*	
H24F	0.5039	0.5812	0.6215	0.027*	
C25B	0.63789 (17)	0.6318 (3)	0.69153 (12)	0.0195 (5)	
H25B	0.6852	0.7126	0.6951	0.023*	
C26B	0.61099 (19)	0.6148 (3)	0.76606 (13)	0.0242 (5)	
H26A	0.5760	0.6990	0.7770	0.036*	
H26B	0.6720	0.6017	0.8041	0.036*	
H26C	0.5677	0.5328	0.7649	0.036*	
C27B	0.69108 (19)	0.5011 (3)	0.67274 (15)	0.0273 (5)	
H27A	0.7462	0.4775	0.7140	0.041*	
H27B	0.7173	0.5192	0.6289	0.041*	
H27C	0.6439	0.4226	0.6632	0.041*	

### Atomic displacement parameters (Å^2^)

**Table:**

	*U*^11^	*U*^22^	*U*^33^	*U*^12^	*U*^13^	*U*^23^
O1A	0.0173 (8)	0.0416 (11)	0.0223 (9)	−0.0053 (8)	0.0042 (7)	−0.0046 (8)
C1A	0.0198 (11)	0.0177 (10)	0.0207 (11)	−0.0026 (9)	0.0069 (9)	−0.0005 (8)
N1A	0.0184 (9)	0.0247 (10)	0.0253 (10)	−0.0018 (8)	0.0079 (8)	−0.0001 (8)
C2A	0.0210 (11)	0.0218 (11)	0.0240 (12)	0.0049 (9)	0.0087 (9)	0.0053 (9)
C3A	0.0287 (14)	0.0415 (16)	0.0249 (13)	0.0109 (12)	0.0124 (10)	0.0111 (11)
C4A	0.0325 (15)	0.063 (2)	0.0299 (14)	0.0213 (15)	0.0167 (12)	0.0167 (14)
C5A	0.0210 (13)	0.061 (2)	0.0428 (17)	0.0150 (14)	0.0167 (12)	0.0300 (15)
C6A	0.0132 (11)	0.0363 (15)	0.0406 (15)	0.0057 (10)	0.0085 (10)	0.0173 (12)
C7A	0.0127 (11)	0.0193 (11)	0.0330 (13)	0.0022 (9)	0.0050 (9)	0.0083 (9)
C8A	0.0123 (10)	0.0137 (10)	0.0285 (12)	0.0013 (8)	0.0038 (9)	0.0010 (9)
C9A	0.0122 (11)	0.0239 (12)	0.0409 (15)	−0.0034 (9)	0.0040 (10)	−0.0065 (11)
C10A	0.0131 (11)	0.0268 (12)	0.0324 (13)	0.0005 (9)	−0.0016 (9)	−0.0082 (10)
C11A	0.0146 (11)	0.0177 (10)	0.0266 (12)	0.0021 (9)	−0.0008 (9)	−0.0087 (9)
C12A	0.0191 (11)	0.0169 (10)	0.0272 (12)	0.0041 (9)	0.0004 (9)	−0.0058 (9)
C13A	0.0210 (12)	0.0317 (13)	0.0233 (12)	0.0020 (10)	0.0034 (9)	−0.0073 (10)
C14A	0.0171 (11)	0.0267 (12)	0.0233 (12)	−0.0015 (9)	0.0021 (9)	−0.0069 (10)
C15A	0.0131 (10)	0.0177 (10)	0.0248 (11)	−0.0001 (8)	0.0022 (8)	−0.0062 (9)
C16A	0.0121 (10)	0.0161 (10)	0.0263 (12)	−0.0014 (8)	0.0021 (8)	−0.0034 (8)
C17A	0.0156 (10)	0.0253 (12)	0.0233 (11)	−0.0061 (9)	0.0049 (8)	−0.0036 (9)
C18A	0.0222 (13)	0.0222 (12)	0.0464 (17)	0.0007 (10)	0.0033 (11)	0.0111 (11)
C19A	0.0215 (12)	0.0184 (11)	0.0308 (14)	0.0040 (9)	0.0009 (10)	−0.0064 (9)
C20A	0.0322 (14)	0.0173 (11)	0.0240 (12)	0.0035 (10)	−0.0048 (10)	−0.0034 (9)
C21A	0.0397 (16)	0.0263 (13)	0.0333 (15)	−0.0116 (12)	−0.0142 (12)	0.0052 (11)
C22A	0.0265 (13)	0.0225 (12)	0.0305 (14)	0.0031 (10)	−0.0054 (10)	0.0008 (10)
C23A	0.042 (2)	0.036 (2)	0.030 (2)	−0.0142 (17)	0.0005 (17)	0.0047 (16)
C24A	0.036 (3)	0.031 (2)	0.027 (2)	−0.0029 (19)	0.0029 (18)	0.0015 (18)
C25A	0.0153 (16)	0.0239 (16)	0.0203 (16)	0.0007 (14)	0.0080 (13)	−0.0069 (14)
C23C	0.041 (2)	0.0281 (19)	0.0241 (18)	−0.0026 (17)	0.0079 (16)	0.0001 (15)
C24C	0.0178 (13)	0.0242 (14)	0.0176 (13)	0.0039 (12)	0.0060 (11)	0.0027 (11)
C25C	0.036 (2)	0.0318 (19)	0.0330 (19)	0.0023 (16)	0.0065 (16)	0.0014 (16)
C26A	0.0529 (15)	0.0336 (13)	0.0274 (12)	0.0046 (12)	0.0009 (11)	−0.0019 (10)
C27A	0.0711 (17)	0.0354 (13)	0.0297 (13)	−0.0002 (13)	0.0092 (12)	0.0034 (11)
O1B	0.0138 (8)	0.0315 (9)	0.0222 (8)	0.0029 (7)	0.0037 (6)	−0.0031 (7)
C1B	0.0194 (11)	0.0167 (10)	0.0158 (10)	−0.0029 (8)	0.0054 (8)	−0.0015 (8)
N1B	0.0136 (9)	0.0205 (9)	0.0198 (9)	0.0000 (7)	0.0049 (7)	−0.0024 (7)
C2B	0.0200 (11)	0.0151 (10)	0.0155 (10)	0.0004 (8)	0.0055 (8)	0.0005 (8)
C3B	0.0216 (12)	0.0244 (11)	0.0163 (10)	0.0010 (9)	0.0071 (9)	−0.0002 (9)
C4B	0.0253 (12)	0.0220 (11)	0.0216 (12)	0.0050 (10)	0.0103 (10)	0.0012 (9)
C5B	0.0206 (12)	0.0250 (12)	0.0267 (12)	0.0034 (10)	0.0107 (10)	0.0013 (9)
C6B	0.0172 (11)	0.0268 (12)	0.0222 (12)	0.0026 (9)	0.0078 (9)	0.0017 (9)
C7B	0.0148 (10)	0.0166 (10)	0.0177 (10)	−0.0002 (8)	0.0039 (8)	0.0015 (8)
C8B	0.0160 (10)	0.0167 (10)	0.0152 (10)	0.0001 (8)	0.0038 (8)	0.0007 (8)
C9B	0.0151 (10)	0.0218 (11)	0.0224 (11)	−0.0009 (9)	0.0042 (8)	−0.0029 (9)
C10B	0.0162 (10)	0.0237 (11)	0.0180 (11)	0.0041 (9)	0.0014 (8)	−0.0025 (9)
C11B	0.0161 (10)	0.0140 (10)	0.0161 (10)	0.0013 (8)	0.0020 (8)	−0.0011 (8)
C12B	0.0235 (11)	0.0143 (10)	0.0167 (10)	0.0030 (9)	0.0043 (8)	−0.0003 (8)
C13B	0.0258 (12)	0.0203 (11)	0.0155 (10)	−0.0027 (9)	0.0078 (9)	−0.0011 (8)
C14B	0.0221 (11)	0.0230 (11)	0.0160 (10)	−0.0067 (9)	0.0073 (8)	−0.0017 (8)
C15B	0.0194 (11)	0.0138 (9)	0.0136 (10)	−0.0024 (8)	0.0034 (8)	−0.0009 (7)
C16B	0.0149 (10)	0.0165 (10)	0.0138 (9)	−0.0009 (8)	0.0039 (7)	−0.0002 (7)
C17B	0.0182 (11)	0.0220 (11)	0.0172 (10)	−0.0029 (9)	0.0060 (9)	−0.0013 (8)
C18B	0.0257 (12)	0.0155 (10)	0.0250 (12)	−0.0008 (9)	0.0091 (10)	0.0029 (9)
C19B	0.0206 (11)	0.0131 (10)	0.0231 (11)	0.0001 (8)	0.0041 (9)	0.0012 (8)
C20B	0.0236 (12)	0.0168 (10)	0.0183 (11)	0.0028 (9)	0.0028 (9)	−0.0012 (8)
C21B	0.0278 (13)	0.0280 (12)	0.0216 (12)	0.0054 (11)	0.0018 (10)	−0.0073 (10)
C22B	0.0344 (14)	0.0190 (11)	0.0166 (11)	0.0018 (10)	0.0045 (9)	−0.0017 (9)
C23B	0.0396 (15)	0.0200 (11)	0.0170 (11)	0.0050 (11)	0.0052 (10)	−0.0013 (9)
C24B	0.0241 (12)	0.0232 (11)	0.0189 (11)	0.0023 (10)	0.0032 (9)	−0.0018 (9)
C25B	0.0167 (10)	0.0220 (11)	0.0197 (11)	−0.0029 (9)	0.0036 (8)	−0.0008 (9)
C26B	0.0266 (13)	0.0277 (12)	0.0186 (11)	−0.0024 (10)	0.0053 (9)	0.0004 (10)
C27B	0.0206 (12)	0.0335 (14)	0.0264 (13)	0.0058 (11)	0.0020 (10)	−0.0035 (11)

### Geometric parameters (Å, º)

**Table:**

O1A—N1A	1.416 (3)	C26A—H26H	0.9543
O1A—H1A	0.87 (3)	C26A—H26I	0.9786
C1A—N1A	1.276 (3)	C27A—H27D	0.9659
C1A—C17A	1.501 (3)	C27A—H27E	0.9622
C1A—C2A	1.498 (3)	C27A—H27F	0.9675
C2A—C3A	1.532 (3)	C27A—H27G	0.9617
C2A—C7A	1.559 (4)	C27A—H27H	0.9675
C2A—H2A	1.0000	C27A—H27I	0.9558
C3A—C4A	1.520 (4)	O1B—N1B	1.423 (2)
C3A—H3A1	0.9900	O1B—H1B	0.87 (3)
C3A—H3A2	0.9900	C1B—N1B	1.279 (3)
C4A—C5A	1.511 (5)	C1B—C2B	1.495 (3)
C4A—H4A1	0.9900	C1B—C17B	1.504 (3)
C4A—H4A2	0.9900	C2B—C3B	1.530 (3)
C5A—C6A	1.533 (4)	C2B—C7B	1.562 (3)
C5A—H5A1	0.9900	C2B—H2B	1.0000
C5A—H5A2	0.9900	C3B—C4B	1.522 (3)
C6A—C7A	1.545 (3)	C3B—H3B1	0.9900
C6A—H6A1	0.9900	C3B—H3B2	0.9900
C6A—H6A2	0.9900	C4B—C5B	1.526 (4)
C7A—C18A	1.539 (3)	C4B—H4B1	0.9900
C7A—C8A	1.549 (3)	C4B—H4B2	0.9900
C8A—C16A	1.539 (3)	C5B—C6B	1.531 (3)
C8A—C9A	1.538 (3)	C5B—H5B1	0.9900
C8A—H8A	1.0000	C5B—H5B2	0.9900
C9A—C10A	1.528 (4)	C6B—C7B	1.545 (3)
C9A—H9A1	0.9900	C6B—H6B1	0.9900
C9A—H9A2	0.9900	C6B—H6B2	0.9900
C10A—C11A	1.532 (3)	C7B—C18B	1.533 (3)
C10A—H10A	0.9900	C7B—C8B	1.559 (3)
C10A—H10B	0.9900	C8B—C9B	1.536 (3)
C11A—C19A	1.536 (3)	C8B—C16B	1.546 (3)
C11A—C15A	1.546 (3)	C8B—H8B	1.0000
C11A—C12A	1.553 (4)	C9B—C10B	1.542 (3)
C12A—C20A	1.542 (3)	C9B—H9B1	0.9900
C12A—C13A	1.554 (3)	C9B—H9B2	0.9900
C12A—H12A	1.0000	C10B—C11B	1.531 (3)
C13A—C14A	1.547 (3)	C10B—H10C	0.9900
C13A—H13A	0.9900	C10B—H10D	0.9900
C13A—H13B	0.9900	C11B—C19B	1.534 (3)
C14A—C15A	1.526 (3)	C11B—C15B	1.545 (3)
C14A—H14A	0.9900	C11B—C12B	1.556 (3)
C14A—H14B	0.9900	C12B—C20B	1.545 (3)
C15A—C16A	1.523 (3)	C12B—C13B	1.557 (3)
C15A—H15A	1.0000	C12B—H12B	1.0000
C16A—C17A	1.539 (3)	C13B—C14B	1.550 (3)
C16A—H16A	1.0000	C13B—H13C	0.9900
C17A—H17A	0.9900	C13B—H13D	0.9900
C17A—H17B	0.9900	C14B—C15B	1.521 (3)
C18A—H18A	0.9800	C14B—H14C	0.9900
C18A—H18B	0.9800	C14B—H14D	0.9900
C18A—H18C	0.9800	C15B—C16B	1.531 (3)
C19A—H19A	0.9800	C15B—H15B	1.0000
C19A—H19B	0.9800	C16B—C17B	1.536 (3)
C19A—H19C	0.9800	C16B—H16B	1.0000
C20A—C21A	1.536 (4)	C17B—H17C	0.9900
C20A—C22A	1.532 (4)	C17B—H17D	0.9900
C20A—H20A	1.0000	C18B—H18D	0.9800
C21A—H21A	0.9800	C18B—H18E	0.9800
C21A—H21B	0.9800	C18B—H18F	0.9800
C21A—H21C	0.9800	C19B—H19D	0.9800
C22A—C23C	1.496 (5)	C19B—H19E	0.9800
C22A—C23A	1.581 (6)	C19B—H19F	0.9800
C22A—H22A	0.9900	C20B—C21B	1.533 (4)
C22A—H22B	0.9900	C20B—C22B	1.541 (3)
C23A—C24A	1.542 (7)	C20B—H20B	1.0000
C23A—H23A	0.9900	C21B—H21D	0.9800
C23A—H23B	0.9900	C21B—H21E	0.9800
C24A—C25A	1.603 (7)	C21B—H21F	0.9800
C24A—H24A	0.9900	C22B—C23B	1.535 (3)
C24A—H24B	0.9900	C22B—H22C	0.9900
C25A—C26A	1.494 (5)	C22B—H22D	0.9900
C25A—C27A	1.582 (5)	C23B—C24B	1.534 (3)
C25A—H25A	1.0000	C23B—H23E	0.9900
C23C—C24C	1.511 (6)	C23B—H23F	0.9900
C23C—H23C	0.9900	C24B—C25B	1.529 (3)
C23C—H23D	0.9900	C24B—H24E	0.9900
C24C—C25C	1.505 (6)	C24B—H24F	0.9900
C24C—H24C	0.9900	C25B—C27B	1.524 (4)
C24C—H24D	0.9900	C25B—C26B	1.527 (3)
C25C—C27A	1.548 (6)	C25B—H25B	1.0000
C25C—C26A	1.618 (6)	C26B—H26A	0.9800
C25C—H25C	1.0000	C26B—H26B	0.9800
C26A—H26D	0.9758	C26B—H26C	0.9800
C26A—H26E	0.9658	C27B—H27A	0.9800
C26A—H26F	0.9514	C27B—H27B	0.9800
C26A—H26G	0.9625	C27B—H27C	0.9800
			
N1A—O1A—H1A	96 (2)	C25C—C26A—H26I	110.3
N1A—C1A—C17A	126.0 (2)	H26D—C26A—H26I	48.9
N1A—C1A—C2A	118.2 (2)	H26E—C26A—H26I	90.9
C17A—C1A—C2A	115.76 (19)	H26F—C26A—H26I	154.8
C1A—N1A—O1A	113.69 (19)	H26G—C26A—H26I	107.7
C1A—C2A—C3A	114.6 (2)	H26H—C26A—H26I	108.6
C1A—C2A—C7A	110.2 (2)	C25C—C27A—H27D	139.1
C3A—C2A—C7A	114.0 (2)	C25A—C27A—H27D	111.2
C1A—C2A—H2A	105.7	C25C—C27A—H27E	77.5
C3A—C2A—H2A	105.7	C25A—C27A—H27E	110.5
C7A—C2A—H2A	105.7	H27D—C27A—H27E	108.7
C4A—C3A—C2A	110.5 (2)	C25C—C27A—H27F	107.4
C4A—C3A—H3A1	109.6	C25A—C27A—H27F	109.3
C2A—C3A—H3A1	109.6	H27D—C27A—H27F	108.4
C4A—C3A—H3A2	109.6	H27E—C27A—H27F	108.7
C2A—C3A—H3A2	109.6	C25C—C27A—H27G	110.6
H3A1—C3A—H3A2	108.1	C25A—C27A—H27G	124.9
C5A—C4A—C3A	110.6 (3)	H27D—C27A—H27G	109.9
C5A—C4A—H4A1	109.5	H27E—C27A—H27G	88.8
C3A—C4A—H4A1	109.5	C25C—C27A—H27H	109.9
C5A—C4A—H4A2	109.5	C25A—C27A—H27H	73.5
C3A—C4A—H4A2	109.5	H27D—C27A—H27H	50.3
H4A1—C4A—H4A2	108.1	H27E—C27A—H27H	155.8
C4A—C5A—C6A	111.9 (2)	H27F—C27A—H27H	91.4
C4A—C5A—H5A1	109.2	H27G—C27A—H27H	108.8
C6A—C5A—H5A1	109.2	C25C—C27A—H27I	108.7
C4A—C5A—H5A2	109.2	C25A—C27A—H27I	121.7
C6A—C5A—H5A2	109.2	H27D—C27A—H27I	61.5
H5A1—C5A—H5A2	107.9	H27E—C27A—H27I	47.6
C5A—C6A—C7A	113.8 (2)	H27F—C27A—H27I	128.3
C5A—C6A—H6A1	108.8	H27G—C27A—H27I	109.5
C7A—C6A—H6A1	108.8	H27H—C27A—H27I	109.2
C5A—C6A—H6A2	108.8	N1B—O1B—H1B	100 (2)
C7A—C6A—H6A2	108.8	N1B—C1B—C2B	118.5 (2)
H6A1—C6A—H6A2	107.7	N1B—C1B—C17B	126.6 (2)
C18A—C7A—C6A	110.1 (2)	C2B—C1B—C17B	114.83 (19)
C18A—C7A—C8A	111.4 (2)	C1B—N1B—O1B	113.34 (19)
C6A—C7A—C8A	109.87 (19)	C1B—C2B—C3B	114.90 (18)
C18A—C7A—C2A	110.3 (2)	C1B—C2B—C7B	109.72 (18)
C6A—C7A—C2A	108.0 (2)	C3B—C2B—C7B	113.36 (19)
C8A—C7A—C2A	107.09 (18)	C1B—C2B—H2B	106.0
C16A—C8A—C9A	109.79 (19)	C3B—C2B—H2B	106.0
C16A—C8A—C7A	112.80 (18)	C7B—C2B—H2B	106.0
C9A—C8A—C7A	115.4 (2)	C4B—C3B—C2B	110.39 (19)
C16A—C8A—H8A	106.0	C4B—C3B—H3B1	109.6
C9A—C8A—H8A	106.0	C2B—C3B—H3B1	109.6
C7A—C8A—H8A	106.0	C4B—C3B—H3B2	109.6
C10A—C9A—C8A	113.1 (2)	C2B—C3B—H3B2	109.6
C10A—C9A—H9A1	109.0	H3B1—C3B—H3B2	108.1
C8A—C9A—H9A1	109.0	C3B—C4B—C5B	110.9 (2)
C10A—C9A—H9A2	109.0	C3B—C4B—H4B1	109.5
C8A—C9A—H9A2	109.0	C5B—C4B—H4B1	109.5
H9A1—C9A—H9A2	107.8	C3B—C4B—H4B2	109.5
C9A—C10A—C11A	112.7 (2)	C5B—C4B—H4B2	109.5
C9A—C10A—H10A	109.0	H4B1—C4B—H4B2	108.1
C11A—C10A—H10A	109.0	C4B—C5B—C6B	111.6 (2)
C9A—C10A—H10B	109.0	C4B—C5B—H5B1	109.3
C11A—C10A—H10B	109.0	C6B—C5B—H5B1	109.3
H10A—C10A—H10B	107.8	C4B—C5B—H5B2	109.3
C10A—C11A—C19A	110.5 (2)	C6B—C5B—H5B2	109.3
C10A—C11A—C15A	107.43 (19)	H5B1—C5B—H5B2	108.0
C19A—C11A—C15A	112.08 (19)	C5B—C6B—C7B	113.79 (19)
C10A—C11A—C12A	117.5 (2)	C5B—C6B—H6B1	108.8
C19A—C11A—C12A	110.1 (2)	C7B—C6B—H6B1	108.8
C15A—C11A—C12A	98.66 (19)	C5B—C6B—H6B2	108.8
C20A—C12A—C11A	120.5 (2)	C7B—C6B—H6B2	108.8
C20A—C12A—C13A	112.1 (2)	H6B1—C6B—H6B2	107.7
C11A—C12A—C13A	103.23 (19)	C18B—C7B—C6B	110.33 (19)
C20A—C12A—H12A	106.7	C18B—C7B—C8B	111.22 (19)
C11A—C12A—H12A	106.7	C6B—C7B—C8B	109.98 (18)
C13A—C12A—H12A	106.7	C18B—C7B—C2B	110.54 (18)
C14A—C13A—C12A	106.6 (2)	C6B—C7B—C2B	106.90 (19)
C14A—C13A—H13A	110.4	C8B—C7B—C2B	107.74 (17)
C12A—C13A—H13A	110.4	C9B—C8B—C16B	110.23 (18)
C14A—C13A—H13B	110.4	C9B—C8B—C7B	113.48 (18)
C12A—C13A—H13B	110.4	C16B—C8B—C7B	113.20 (17)
H13A—C13A—H13B	108.6	C9B—C8B—H8B	106.5
C15A—C14A—C13A	103.35 (18)	C16B—C8B—H8B	106.5
C15A—C14A—H14A	111.1	C7B—C8B—H8B	106.5
C13A—C14A—H14A	111.1	C8B—C9B—C10B	113.74 (19)
C15A—C14A—H14B	111.1	C8B—C9B—H9B1	108.8
C13A—C14A—H14B	111.1	C10B—C9B—H9B1	108.8
H14A—C14A—H14B	109.1	C8B—C9B—H9B2	108.8
C16A—C15A—C14A	119.35 (19)	C10B—C9B—H9B2	108.8
C16A—C15A—C11A	114.9 (2)	H9B1—C9B—H9B2	107.7
C14A—C15A—C11A	104.40 (18)	C11B—C10B—C9B	112.10 (18)
C16A—C15A—H15A	105.7	C11B—C10B—H10C	109.2
C14A—C15A—H15A	105.7	C9B—C10B—H10C	109.2
C11A—C15A—H15A	105.7	C11B—C10B—H10D	109.2
C15A—C16A—C8A	108.72 (18)	C9B—C10B—H10D	109.2
C15A—C16A—C17A	109.77 (19)	H10C—C10B—H10D	107.9
C8A—C16A—C17A	111.73 (19)	C10B—C11B—C19B	111.40 (19)
C15A—C16A—H16A	108.9	C10B—C11B—C15B	106.94 (18)
C8A—C16A—H16A	108.9	C19B—C11B—C15B	111.59 (18)
C17A—C16A—H16A	108.9	C10B—C11B—C12B	116.93 (18)
C1A—C17A—C16A	113.28 (19)	C19B—C11B—C12B	109.29 (19)
C1A—C17A—H17A	108.9	C15B—C11B—C12B	100.14 (17)
C16A—C17A—H17A	108.9	C20B—C12B—C11B	118.77 (19)
C1A—C17A—H17B	108.9	C20B—C12B—C13B	111.80 (18)
C16A—C17A—H17B	108.9	C11B—C12B—C13B	102.94 (17)
H17A—C17A—H17B	107.7	C20B—C12B—H12B	107.6
C7A—C18A—H18A	109.5	C11B—C12B—H12B	107.6
C7A—C18A—H18B	109.5	C13B—C12B—H12B	107.6
H18A—C18A—H18B	109.5	C14B—C13B—C12B	106.93 (18)
C7A—C18A—H18C	109.5	C14B—C13B—H13C	110.3
H18A—C18A—H18C	109.5	C12B—C13B—H13C	110.3
H18B—C18A—H18C	109.5	C14B—C13B—H13D	110.3
C11A—C19A—H19A	109.5	C12B—C13B—H13D	110.3
C11A—C19A—H19B	109.5	H13C—C13B—H13D	108.6
H19A—C19A—H19B	109.5	C15B—C14B—C13B	104.03 (18)
C11A—C19A—H19C	109.5	C15B—C14B—H14C	111.0
H19A—C19A—H19C	109.5	C13B—C14B—H14C	111.0
H19B—C19A—H19C	109.5	C15B—C14B—H14D	111.0
C21A—C20A—C22A	110.9 (2)	C13B—C14B—H14D	111.0
C21A—C20A—C12A	111.5 (2)	H14C—C14B—H14D	109.0
C22A—C20A—C12A	109.1 (2)	C14B—C15B—C16B	118.65 (18)
C21A—C20A—H20A	108.4	C14B—C15B—C11B	104.42 (17)
C22A—C20A—H20A	108.4	C16B—C15B—C11B	112.97 (18)
C12A—C20A—H20A	108.4	C14B—C15B—H15B	106.7
C20A—C21A—H21A	109.5	C16B—C15B—H15B	106.7
C20A—C21A—H21B	109.5	C11B—C15B—H15B	106.7
H21A—C21A—H21B	109.5	C15B—C16B—C17B	111.56 (18)
C20A—C21A—H21C	109.5	C15B—C16B—C8B	108.96 (17)
H21A—C21A—H21C	109.5	C17B—C16B—C8B	111.85 (18)
H21B—C21A—H21C	109.5	C15B—C16B—H16B	108.1
C23C—C22A—C20A	113.8 (3)	C17B—C16B—H16B	108.1
C20A—C22A—C23A	118.2 (3)	C8B—C16B—H16B	108.1
C23C—C22A—H22A	84.1	C1B—C17B—C16B	109.29 (18)
C20A—C22A—H22A	107.8	C1B—C17B—H17C	109.8
C23A—C22A—H22A	107.8	C16B—C17B—H17C	109.8
C23C—C22A—H22B	131.0	C1B—C17B—H17D	109.8
C20A—C22A—H22B	107.8	C16B—C17B—H17D	109.8
C23A—C22A—H22B	107.8	H17C—C17B—H17D	108.3
H22A—C22A—H22B	107.1	C7B—C18B—H18D	109.5
C24A—C23A—C22A	125.9 (6)	C7B—C18B—H18E	109.5
C24A—C23A—H23A	105.8	H18D—C18B—H18E	109.5
C22A—C23A—H23A	105.8	C7B—C18B—H18F	109.5
C24A—C23A—H23B	105.8	H18D—C18B—H18F	109.5
C22A—C23A—H23B	105.8	H18E—C18B—H18F	109.5
H23A—C23A—H23B	106.2	C11B—C19B—H19D	109.5
C23A—C24A—C25A	116.9 (6)	C11B—C19B—H19E	109.5
C23A—C24A—H24A	108.1	H19D—C19B—H19E	109.5
C25A—C24A—H24A	108.1	C11B—C19B—H19F	109.5
C23A—C24A—H24B	108.1	H19D—C19B—H19F	109.5
C25A—C24A—H24B	108.1	H19E—C19B—H19F	109.5
H24A—C24A—H24B	107.3	C21B—C20B—C22B	109.9 (2)
C26A—C25A—C27A	108.5 (3)	C21B—C20B—C12B	112.52 (19)
C26A—C25A—C24A	100.3 (5)	C22B—C20B—C12B	111.13 (19)
C27A—C25A—C24A	100.7 (4)	C21B—C20B—H20B	107.7
C26A—C25A—H25A	115.1	C22B—C20B—H20B	107.7
C27A—C25A—H25A	115.1	C12B—C20B—H20B	107.7
C24A—C25A—H25A	115.1	C20B—C21B—H21D	109.5
C27A—C25A—H26I	117.5	C20B—C21B—H21E	109.5
C24A—C25A—H26I	128.6	H21D—C21B—H21E	109.5
H25A—C25A—H26I	79.4	C20B—C21B—H21F	109.5
C22A—C23C—C24C	108.8 (4)	H21D—C21B—H21F	109.5
C22A—C23C—H23C	109.9	H21E—C21B—H21F	109.5
C24C—C23C—H23C	109.9	C23B—C22B—C20B	112.4 (2)
C22A—C23C—H23D	109.9	C23B—C22B—H22C	109.1
C24C—C23C—H23D	109.9	C20B—C22B—H22C	109.1
H23C—C23C—H23D	108.3	C23B—C22B—H22D	109.1
C25C—C24C—C23C	122.0 (4)	C20B—C22B—H22D	109.1
C25C—C24C—H24C	106.8	H22C—C22B—H22D	107.8
C23C—C24C—H24C	106.8	C24B—C23B—C22B	116.7 (2)
C25C—C24C—H24D	106.8	C24B—C23B—H23E	108.1
C23C—C24C—H24D	106.8	C22B—C23B—H23E	108.1
H24C—C24C—H24D	106.7	C24B—C23B—H23F	108.1
C24C—C25C—C27A	108.1 (4)	C22B—C23B—H23F	108.1
C24C—C25C—C26A	109.9 (4)	H23E—C23B—H23F	107.3
C27A—C25C—C26A	104.2 (3)	C25B—C24B—C23B	116.1 (2)
C24C—C25C—H25C	111.4	C25B—C24B—H24E	108.3
C27A—C25C—H25C	111.4	C23B—C24B—H24E	108.3
C26A—C25C—H25C	111.4	C25B—C24B—H24F	108.3
C25A—C26A—H26D	110.2	C23B—C24B—H24F	108.3
C25C—C26A—H26D	135.7	H24E—C24B—H24F	107.4
C25A—C26A—H26E	109.9	C27B—C25B—C26B	110.4 (2)
C25C—C26A—H26E	111.5	C27B—C25B—C24B	112.4 (2)
H26D—C26A—H26E	107.7	C26B—C25B—C24B	110.4 (2)
C25A—C26A—H26F	110.4	C27B—C25B—H25B	107.8
C25C—C26A—H26F	76.3	C26B—C25B—H25B	107.8
H26D—C26A—H26F	108.9	C24B—C25B—H25B	107.8
H26E—C26A—H26F	109.7	C25B—C26B—H26A	109.5
C25A—C26A—H26G	120.6	C25B—C26B—H26B	109.5
C25C—C26A—H26G	110.4	H26A—C26B—H26B	109.5
H26D—C26A—H26G	113.3	C25B—C26B—H26C	109.5
H26F—C26A—H26G	91.6	H26A—C26B—H26C	109.5
C25A—C26A—H26H	126.2	H26B—C26B—H26C	109.5
C25C—C26A—H26H	110.0	C25B—C27B—H27A	109.5
H26D—C26A—H26H	60.9	C25B—C27B—H27B	109.5
H26E—C26A—H26H	123.5	H27A—C27B—H27B	109.5
H26F—C26A—H26H	48.0	C25B—C27B—H27C	109.5
H26G—C26A—H26H	109.8	H27A—C27B—H27C	109.5
C25A—C26A—H26I	74.0	H27B—C27B—H27C	109.5
			
C17A—C1A—N1A—O1A	1.6 (3)	C27A—C25A—C26A—C25C	−64.1 (4)
C2A—C1A—N1A—O1A	178.6 (2)	C24A—C25A—C26A—C25C	41.1 (4)
N1A—C1A—C2A—C3A	−2.9 (3)	C24C—C25C—C26A—C25A	−51.6 (4)
C17A—C1A—C2A—C3A	174.4 (2)	C27A—C25C—C26A—C25A	64.0 (4)
N1A—C1A—C2A—C7A	127.3 (2)	C24C—C25C—C27A—C25A	58.9 (4)
C17A—C1A—C2A—C7A	−55.4 (3)	C26A—C25C—C27A—C25A	−58.0 (4)
C1A—C2A—C3A—C4A	−175.2 (2)	C26A—C25A—C27A—C25C	69.9 (4)
C7A—C2A—C3A—C4A	56.6 (3)	C24A—C25A—C27A—C25C	−34.9 (5)
C2A—C3A—C4A—C5A	−57.0 (3)	C2B—C1B—N1B—O1B	−179.16 (19)
C3A—C4A—C5A—C6A	56.6 (3)	C17B—C1B—N1B—O1B	−2.4 (3)
C4A—C5A—C6A—C7A	−55.2 (3)	N1B—C1B—C2B—C3B	−12.9 (3)
C5A—C6A—C7A—C18A	−69.5 (3)	C17B—C1B—C2B—C3B	169.95 (19)
C5A—C6A—C7A—C8A	167.4 (2)	N1B—C1B—C2B—C7B	116.2 (2)
C5A—C6A—C7A—C2A	51.0 (3)	C17B—C1B—C2B—C7B	−60.9 (2)
C1A—C2A—C7A—C18A	−62.4 (3)	C1B—C2B—C3B—C4B	−174.5 (2)
C3A—C2A—C7A—C18A	68.1 (3)	C7B—C2B—C3B—C4B	58.2 (3)
C1A—C2A—C7A—C6A	177.30 (19)	C2B—C3B—C4B—C5B	−56.1 (3)
C3A—C2A—C7A—C6A	−52.3 (3)	C3B—C4B—C5B—C6B	54.9 (3)
C1A—C2A—C7A—C8A	59.0 (2)	C4B—C5B—C6B—C7B	−55.6 (3)
C3A—C2A—C7A—C8A	−170.53 (19)	C5B—C6B—C7B—C18B	−66.5 (3)
C18A—C7A—C8A—C16A	61.3 (2)	C5B—C6B—C7B—C8B	170.40 (19)
C6A—C7A—C8A—C16A	−176.5 (2)	C5B—C6B—C7B—C2B	53.7 (3)
C2A—C7A—C8A—C16A	−59.5 (2)	C1B—C2B—C7B—C18B	−65.2 (2)
C18A—C7A—C8A—C9A	−66.1 (3)	C3B—C2B—C7B—C18B	64.7 (2)
C6A—C7A—C8A—C9A	56.2 (3)	C1B—C2B—C7B—C6B	174.70 (18)
C2A—C7A—C8A—C9A	173.19 (19)	C3B—C2B—C7B—C6B	−55.4 (2)
C16A—C8A—C9A—C10A	55.5 (3)	C1B—C2B—C7B—C8B	56.5 (2)
C7A—C8A—C9A—C10A	−175.6 (2)	C3B—C2B—C7B—C8B	−173.54 (18)
C8A—C9A—C10A—C11A	−55.1 (3)	C18B—C7B—C8B—C9B	−59.9 (2)
C9A—C10A—C11A—C19A	−70.1 (3)	C6B—C7B—C8B—C9B	62.6 (2)
C9A—C10A—C11A—C15A	52.5 (3)	C2B—C7B—C8B—C9B	178.76 (18)
C9A—C10A—C11A—C12A	162.4 (2)	C18B—C7B—C8B—C16B	66.7 (2)
C10A—C11A—C12A—C20A	76.5 (3)	C6B—C7B—C8B—C16B	−170.81 (19)
C19A—C11A—C12A—C20A	−51.2 (3)	C2B—C7B—C8B—C16B	−54.6 (2)
C15A—C11A—C12A—C20A	−168.6 (2)	C16B—C8B—C9B—C10B	52.2 (2)
C10A—C11A—C12A—C13A	−157.6 (2)	C7B—C8B—C9B—C10B	−179.69 (19)
C19A—C11A—C12A—C13A	74.8 (2)	C8B—C9B—C10B—C11B	−53.8 (3)
C15A—C11A—C12A—C13A	−42.7 (2)	C9B—C10B—C11B—C19B	−66.9 (2)
C20A—C12A—C13A—C14A	154.2 (2)	C9B—C10B—C11B—C15B	55.2 (2)
C11A—C12A—C13A—C14A	23.0 (2)	C9B—C10B—C11B—C12B	166.40 (19)
C12A—C13A—C14A—C15A	6.7 (3)	C10B—C11B—C12B—C20B	80.1 (3)
C13A—C14A—C15A—C16A	−164.5 (2)	C19B—C11B—C12B—C20B	−47.6 (3)
C13A—C14A—C15A—C11A	−34.4 (2)	C15B—C11B—C12B—C20B	−164.89 (19)
C10A—C11A—C15A—C16A	−56.6 (3)	C10B—C11B—C12B—C13B	−155.79 (19)
C19A—C11A—C15A—C16A	65.0 (3)	C19B—C11B—C12B—C13B	76.5 (2)
C12A—C11A—C15A—C16A	−179.06 (19)	C15B—C11B—C12B—C13B	−40.8 (2)
C10A—C11A—C15A—C14A	170.8 (2)	C20B—C12B—C13B—C14B	150.24 (19)
C19A—C11A—C15A—C14A	−67.6 (3)	C11B—C12B—C13B—C14B	21.6 (2)
C12A—C11A—C15A—C14A	48.3 (2)	C12B—C13B—C14B—C15B	6.7 (2)
C14A—C15A—C16A—C8A	−175.63 (19)	C13B—C14B—C15B—C16B	−159.81 (19)
C11A—C15A—C16A—C8A	59.2 (2)	C13B—C14B—C15B—C11B	−33.0 (2)
C14A—C15A—C16A—C17A	−53.1 (3)	C10B—C11B—C15B—C14B	168.70 (18)
C11A—C15A—C16A—C17A	−178.32 (19)	C19B—C11B—C15B—C14B	−69.3 (2)
C9A—C8A—C16A—C15A	−55.6 (2)	C12B—C11B—C15B—C14B	46.3 (2)
C7A—C8A—C16A—C15A	174.08 (19)	C10B—C11B—C15B—C16B	−61.0 (2)
C9A—C8A—C16A—C17A	−176.9 (2)	C19B—C11B—C15B—C16B	61.0 (2)
C7A—C8A—C16A—C17A	52.8 (3)	C12B—C11B—C15B—C16B	176.61 (18)
N1A—C1A—C17A—C16A	−135.3 (2)	C14B—C15B—C16B—C17B	−51.9 (3)
C2A—C1A—C17A—C16A	47.7 (3)	C11B—C15B—C16B—C17B	−174.53 (18)
C15A—C16A—C17A—C1A	−165.31 (19)	C14B—C15B—C16B—C8B	−175.85 (19)
C8A—C16A—C17A—C1A	−44.6 (3)	C11B—C15B—C16B—C8B	61.5 (2)
C11A—C12A—C20A—C21A	−55.6 (3)	C9B—C8B—C16B—C15B	−54.5 (2)
C13A—C12A—C20A—C21A	−177.4 (2)	C7B—C8B—C16B—C15B	177.24 (17)
C11A—C12A—C20A—C22A	−178.5 (2)	C9B—C8B—C16B—C17B	−178.27 (19)
C13A—C12A—C20A—C22A	59.8 (3)	C7B—C8B—C16B—C17B	53.4 (2)
C21A—C20A—C22A—C23C	48.0 (4)	N1B—C1B—C17B—C16B	−119.6 (3)
C12A—C20A—C22A—C23C	171.2 (3)	C2B—C1B—C17B—C16B	57.2 (3)
C21A—C20A—C22A—C23A	79.0 (4)	C15B—C16B—C17B—C1B	−173.77 (18)
C12A—C20A—C22A—C23A	−157.8 (3)	C8B—C16B—C17B—C1B	−51.4 (2)
C23C—C22A—C23A—C24A	−82.6 (9)	C11B—C12B—C20B—C21B	−55.1 (3)
C20A—C22A—C23A—C24A	−170.8 (5)	C13B—C12B—C20B—C21B	−174.8 (2)
C22A—C23A—C24A—C25A	90.4 (8)	C11B—C12B—C20B—C22B	−178.8 (2)
C23A—C24A—C25A—C26A	78.9 (7)	C13B—C12B—C20B—C22B	61.5 (2)
C23A—C24A—C25A—C27A	−169.8 (6)	C21B—C20B—C22B—C23B	73.3 (3)
C20A—C22A—C23C—C24C	−177.1 (3)	C12B—C20B—C22B—C23B	−161.5 (2)
C23A—C22A—C23C—C24C	77.1 (7)	C20B—C22B—C23B—C24B	−171.1 (2)
C22A—C23C—C24C—C25C	−156.5 (4)	C22B—C23B—C24B—C25B	91.1 (3)
C23C—C24C—C25C—C27A	−151.5 (4)	C23B—C24B—C25B—C27B	−71.7 (3)
C23C—C24C—C25C—C26A	−38.4 (6)	C23B—C24B—C25B—C26B	164.5 (2)
